# Prognostic model of patients with acute pancreatitis based on prothrombin time—international normalized ratio to albumin ratio: a single-center retrospective study

**DOI:** 10.7717/peerj.21415

**Published:** 2026-06-16

**Authors:** Jun Zhou, Yuexinzi Jin, Litao Zhang, Zhenzhen Cai, Jingping Liu

**Affiliations:** 1 Department of Laboratory Medicine, The First Affiliated Hospital with Nanjing Medical University, Nanjing, Jiangsu, China; 2 Branch of National Clinical Research Center for Laboratory Medicine, Nanjing, Jiangsu, China

**Keywords:** Acute pancreatitis, Model, Prognosis, Prothrombin time-international normalized ratio to albumin ratio

## Abstract

**Background:**

The prothrombin time-international normalized ratio to albumin ratio (PTAR) serves as a prognostic biomarker in several diseases, yet its role in predicting the mortality of acute pancreatitis (AP) remains unclear. This study aimed to investigate the association between PTAR and the 30-day outcomes in AP patients.

**Methods:**

We retrospectively analysed data from 965 patients hospitalized with AP between January 2017 and December 2019, collecting baseline clinical characteristics and laboratory data. Using logistic regression analysis and the least absolute shrinkage and selection operator regression, we developed a prognostic nomogram based on PTAR. The nomogram’s predictive performance and calibration were assessed *via* receiver operating characteristic curve analysis and calibration plots, respectively. Bootstrap technology was used for internal validation.

**Results:**

Among the 965 patients, 43 (4.5%) died within 30 days. Multivariate analysis identified seven risk factors independently associated with 30-day mortality of patients with AP: Age, neutrophil percentage, platelets, fibrinogen, aspartate aminotransferase, blood urea nitrogen, and PTAR. The model incorporating these factors, including PTAR, demonstrated excellent discrimination and calibration ability in assessing 30-day mortality (area under the curve (AUC) = 0.914, 95% confidence interval (CI) [0.877–0.951]). Kaplan-Meier survival curve analysis showed that AP patients with a nomogram value >0.082 had a significantly higher risk of 30-day mortality (*P* < 0.001).

**Conclusions:**

PTAR holds clinical value in evaluating the 30-day prognosis of AP patients. The PTAR-based model could be useful independently or as a complement to conventional measures to support clinical decision-making.

## Introduction

Acute pancreatitis (AP) is a common gastrointestinal emergency characterized by premature activation of pancreatic enzymes, leading to autodigestion of pancreatic tissue and potential systemic organ dysfunction (Boxhoorn et al., 2020). Globally, its annual incidence ranges from 13 to 49 per 100,000 individuals, with a mortality rate of approximately two per 100,000 person-years, both exhibiting an increasing trend (Trikudanathan et al., 2024). AP exhibits considerable heterogeneity in disease severity and prognosis. While most patients experience mild, self-limiting symptoms that resolve within 1 to 2 weeks, approximately 20% progress to moderate or severe disease (Tenner et al., 2024). Severe AP can lead to complications such as pancreatic necrosis or multiple organ failure, with mortality rates reaching 30%. Mortality further increases to 30–50% when complicated by infected pancreatic necrosis (IPN). The etiology of AP is diverse (Mederos, Reber & Girgis, 2021), and the most prevalent causative factors in China include gallstones, hypertriglyceridemia, and alcohol. Although recent advances in therapeutic approaches, including minimally invasive surgery and goal-directed fluid resuscitation, have significantly improved patient outcomes, the early and precise identification of high-risk individuals remains a critical clinical challenge.

Currently, employed clinical scoring systems for predicting AP severity, including Ranson criteria, Acute Physiology and Chronic Health Evaluation II (APACHE II), Bedside Index of Severity in Acute Pancreatitis (BISAP), Marshall score, and modified CT severity index (MCTSI), exhibit inherent limitations (Zhou et al., 2019). The Ranson criteria can only be assessed following 48 h of inpatient observation. While the APACHE II score has demonstrated higher accuracy, its complexity and temporal constraints compromise its clinical utility. Conversely, the BISAP score fails to distinguish transient from persistent organ failure and lacks adequate sensitivity for detecting infectious complications, thereby inadequately addressing clinical demands. Systemic inflammation biomarkers such as C-reactive protein (CRP) offer limited diagnostic and prognostic value in certain contexts, particularly in non-infectious AP. Interleukin-6 (IL-6) can predict severity but is constrained by cost and assay complexity. The triglyceride-glucose (TyG) index, reflecting insulin resistance, is associated with disease severity and poor prognosis in AP, yet its predictive performance varies significantly across different etiologies. Therefore, to help clinicians identify high-risk patients at an earlier stage, and consequently improve the survival of these patients and reduce the economic burden, it is urgent to establish a more objective and effective prognostic model that can accurately predict the severity and mortality of patients with AP. The prothrombin time-international normalized ratio to albumin ratio (PTAR) is a recently developed objective scoring system initially established by Haruki et al. (2018). In the retrospective study, PTAR was demonstrated to be a robust prognostic predictor for patients who underwent hepatocellular carcinoma resection and could additionally evaluate postoperative hepatic functional reserve. Several studies also showed that PTAR could predict short-term mortality in hepatitis B virus-related cirrhotic patients (Sheng et al., 2023; Gao et al., 2019; Cai et al., 2021).

Currently, little is known about the ability of PTAR to discriminate prognosis in AP. Considering the accessibility, simplicity, and cost-effectiveness of blood biomarkers, this study aimed to develop and internally validate a non-invasive model based on PTAR and other routine laboratory parameters to assess its value in predicting 30-day mortality in patients with AP.

## Materials and Methods

### Patients

The cohort study was conducted in the First Affiliated Hospital with Nanjing Medical University from January 2017 to December 2019 retrospectively. Currently, the clinical diagnosis of AP necessitates meeting two of the following three criteria: (I) Abdominal pain consistent with AP; (II) serum amylase and/or lipase greater than three times the upper limit of normal; and/or (III) characteristic findings from abdominal imaging (Tenner et al., 2024). The blood samples were collected for examination of blood routine and related biochemical indicators within 24 h after onset of AP. Exclusion criteria: (I) Age < 18 years; (II) active malignant tumors; (III) patients with other infectious disease or additional inflammatory disease other than acute pancreatitis; (IV) patients receiving surgical procedure within 3 months prior to the study period; and (V) without complete laboratory data within 24 h of AP onset and incomplete or missing clinical data. At last, 965 patients diagnosed with AP were enrolled in this research. The study protocol was approved by the Ethics Committee of the First Affiliated Hospital with Nanjing Medical University (Approval number: 2023-SR-043) and was conducted in accordance with the Declaration of Helsinki. The requirement for informed consent was waived by the ethics committee due to the use of anonymized data.

### Data collection and follow-up

Admission clinical characteristics and laboratory parameters of the patients were sourced from the electronic medical records. (I) Demographic variables: sex, age, BMI, smoking, and drinking history. (II) Clinical data: etiology, medical history (hypertriglyceridemia, coronary heart disease, chronic renal failure, and diabetes), vital signs (body temperature, heart rate, respiratory rate, systolic blood pressure, diastolic blood pressure, 24 h urine excretion volume, *etc*.), scoring systems variables (APACHE II score, Marshall score, and Ranson score). (III) Blood tests. The Sysmex XN series automated hematology analyzer (Sysmex, Japan) and Sysmex CS5100 automated analyzer (Sysmex, Kobe, Japan) were used to evaluate complete blood count and coagulation function, respectively. Serum alpha fetoprotein (AFP) and carbohydrate antigen 199 (CA199) were tested with Cobas e602 automated analyzer (Roche, Penzberg, Germany). Biochemical indexes were measured using Beckman Coulter AU5800 analyzer (Beckman Coulter, Brea, CA, USA). The formula for calculating PTAR was prothrombin time-international normalized ratio (PT-INR) divided by albumin (ALB). Patients were followed up for 30 days and divided into survival and nonsurvival groups according to their clinical outcome.

### Statistical analysis

All statistical analyses were performed using SPSS (version 21) and R software (Version 4.3.1; R Core Team, 2023). Quantitative variables, presented as mean ± standard deviation or median (interquartile range), were compared between the two groups using the independent sample t-test or Mann-Whitney U test. Differences in qualitative data were compared using the chi-squared test. Univariate and multivariate regression analyses were used to identify independent predictors and develop a predictive model for the prognosis of patients with AP. The performance of the model was evaluated using receiver operating characteristic (ROC) curves, calibration curves and decision curve analysis (DCA). Positive predictive value (PPV) and negative predictive value (NPV) were calculated alongside sensitivity and specificity. The internal validation was performed using the 1,000 bootstrap method. Comparisons between AUCs were performed using the De-Long test. Youden’s index was used to determine the optimal cut-off point. Kaplan-Meir (K-M) analysis with log-rank test was used to compare survival distributions. Univariable Cox proportional hazards regression was performed to estimate hazard ratios (HRs) with 95% confidence intervals (CIs) for time-to-event outcomes. For variables exhibiting less than 5% missing data, we utilized the random forest algorithm for imputation. Variables with missing rates of 5% or more were excluded from the final analysis to maintain the study’s reliability and rigor. Two-sided *P* < 0.05 in the statistical test was considered statistically significant.

## Results

### Patient characteristics

All-cause mortality within 30 days was observed in 4.5% of 965 patients with AP (581 males and 384 females). We divided the patients into two groups based on whether they survived. The demographics and baseline clinical characteristics of the patients are summarized in Table 1. No significant difference in the distribution of variables between the survivor and nonsurvivor groups were observed except for age, chronic renal failure history, vital signs, and scoring systems variables. It was found that the nonsurvivors had higher C-reactive protein (CRP), white blood cell (WBC), neutrophil (NE) count, NE percentage, red cell distribution width (RDW), mean platelet volume (MPV), prothrombin time (PT), international normalized ratio (PT-INR), activated partial thromboplastin time (APTT), thrombin time (TT), D-dimer, CA199, aspartate aminotransferase (AST), alkaline phosphatase (ALP), lactate dehydrogenase (LDH), creatine kinase (CK), direct bilirubin (DBIL), blood urea nitrogen (UREA), creatinine (CREA), sodium (Na), chlorine (Cl), urinary amylase (uAMY), and PTAR than the survivors (Table 2). Survivors had higher lymphocyte (LY) count, LY percentage, red blood cell (RBC), hemoglobin (HGB), hematocrit (HCT), platelets (PLT), thrombocytocrit (PCT), fibrinogen (FIB), total cholesterol (TC), total protein (TP), ALB, globular proteins (GLB), and Ca (calcium) than the nonsurvivors (all *P* < 0.05). No statistical differences were found in other laboratory characteristics (*i.e*., monocyte (MO) count, MO percentage, mean corpuscular volume (MCV), mean corpuscular hemoglobin (MCH), mean corpuscular hemoglobin concentration (MCHC), platelet distribution width (PDW), AFP, alanine aminotransferase (ALT), γ-glutamyl transpeptidase (GGT), total bilirubin (TB), indirect bilirubin (IBIL), triglycerides (TG), A/G, potassium (K), glucose (GLU), uric acid (UA), and serum amylase (AMY) level, all *P >* 0.05).

**Table 1:** Demographics and baseline clinical characteristics of the study cohort stratified by survivors or not.

Characteristic	Survivor (*n* = 922)	Nonsurvivor (*n* = 43)	*P*
**Sex (male), *n***	553 (60.0%)	28 (65%)	0.501
**Age (years)**	49 (39, 63)	67 (52, 80)	<0.001
**BMI (kg/m** ^**2**^ **)**	25.01 (23.64, 26.83)	25.27 (24.44, 26.51)	0.106
**Alcohol history, *n***	206 (22.3%)	8 (19%)	0.564
**Smoking history, *n***	175 (19.0%)	7 (16%)	0.658
**Etiology of pancreatitis**			
Gallstone, *n*	311 (33.7%)	14 (33%)	0.874
Cholecystitis/cholangitis, *n*	234 (25.4%)	9 (21%)	0.512
Alcoholic, *n*	103 (11.2%)	3 (7%)	0.390
Others (hyperlipidemia, DM, surgery), *n*	51 (5.5%)	2 (5%)	0.805
Unknown, *n*	321 (34.8%)	18 (42%)	0.345
**Past medical history**			
Hypertriglyceridemia, *n*	278 (30.2%)	18 (42%)	0.104
Coronary heart disease, *n*	27 (2.9%)	3 (7%)	0.145
Chronic renal failure, *n*	25 (2.7%)	8 (19%)	<0.001
Diabetes, *n*	162 (17.6%)	7 (16%)	0.828
**Vital signs**			
Body temperature (°C)	36.90 (36.60, 37.30)	37.10 (36.80, 37.70)	0.016
Heart rate (times/min)	81 (77, 98)	100 (84, 118)	<0.001
Respiratory rate (times/min)	18.0 (17.0, 19.0)	18.0 (18.0, 21.5)	0.005
Systolic blood pressure (mmHg)	128 (117, 139)	120 (109, 133)	0.017
Diastolic blood pressure (mmHg)	79 (72, 86)	75 (70, 84)	0.037
24 h urine excretion volume (mL)	1,837 (1,716, 1,954)	1,539 (945, 1,982)	<0.001
Length of hospital stay (d)	9 (7, 14)	13 (6, 19)	0.301
Pancreatic necrosis, *n*	173 (18.8%)	22 (51%)	<0.001
Pleural effusion, *n*	474 (51.4%)	36 (84%)	<0.001
Supplemental oxygen, *n*	266 (28.9%)	36 (84%)	<0.001
**APACHE II score**	5.0 (3.0, 8.0)	11.0 (7.5, 14.0)	<0.001
**Improved Marshall score**	2.00 (1.00, 3.00)	4.00 (2.00, 6.00)	<0.001
**Ranson score**	1.00 (0.00, 2.00)	2.00 (1.00, 3.00)	<0.001

**Note:**

Data are expressed as number, mean ± standard deviation, or median (interquartile range). Abbreviations: BMI, Body mass index; APACHE II, Acute Physiology and Chronic Health Evaluation II Score.

**Table 2:** Laboratory parameters at baseline of the study cohort stratified by survivors or not.

Characteristic	Survivor (*n* = 922)	Nonsurvivor (*n* = 43)	*P*
**CRP (mg/L)**	67 (37, 90)	77 (65, 90)	0.016
**WBC (×10** ^**9**^ **/L)**	10.4 (7.1, 13.7)	13.5 (10.4, 15.8)	0.001
**LY count (×10** ^**9**^ **/L)**	1.11 (0.79, 1.50)	0.87 (0.61, 1.27)	0.017
**MO count (×10** ^**9**^ **/L)**	0.57 (0.39, 0.80)	0.62 (0.41, 0.94)	0.341
**NE count (×10** ^**9**^ **/L)**	8.5 (5.3, 11.7)	11.6 (8.6, 13.9)	<0.001
**LY percentage (%)**	11 (7, 17)	7 (4, 11)	<0.001
**MO percentage (%)**	5.90 (4.50, 7.60)	5.20 (4.16, 6.70)	0.079
**NE percentage (%)**	82 (74, 87)	88 (82, 91)	<0.001
**RBC (×10** ^**12**^ **/L)**	4.29 (3.84, 4.74)	4.06 (3.28, 4.54)	0.010
**HGB (g/L)**	130 ± 22	119 ± 26	0.007
**HCT (%)**	39 (35, 43)	37 (29, 41)	0.015
**MCV (fL)**	90.1 (87.2, 92.9)	90.1 (87.9, 94.5)	0.265
**MCH (pg)**	30.30 (29.40, 31.40)	30.20 (29.35, 31.40)	0.993
**MCHC (g/L)**	336 (330, 344)	332 (327, 340)	0.057
**RDW (%)**	13.30 (12.70, 14.10)	13.96 (13.25, 15.60)	<0.001
**PLT (×10** ^**9**^ **/L)**	187 (146, 236)	151 (101, 173)	<0.001
**PCT (%)**	0.20 (0.16, 0.25)	0.16 (0.13, 0.20)	<0.001
**MPV (fL)**	11.00 (10.10, 11.90)	11.40 (10.80, 12.40)	0.005
**PDW (%)**	14.30 (12.20, 16.70)	14.50 (12.75, 16.85)	0.570
**PT (s)**	13.10 (12.50, 14.00)	14.90 (13.30, 16.90)	<0.001
**PT-INR**	1.14 (1.09, 1.22)	1.30 (1.16, 1.50)	<0.001
**APTT (s)**	28.6 (26.4, 31.3)	33.4 (28.9, 39.4)	<0.001
**FIB (g/L)**	4.47 (3.22, 6.37)	4.03 (2.17, 5.27)	0.010
**TT (s)**	17.00 (16.10, 18.10)	17.70 (17.10, 20.25)	<0.001
**D-dimer (mg/L)**	2.19 (1.01, 4.25)	4.45 (2.70, 8.07)	<0.001
**AFP (ng/mL)**	2.26 (1.61, 3.17)	2.51 (1.84, 4.69)	0.148
**CA199 (U/mL)**	22 (12, 38)	50 (23, 81)	<0.001
**ALT (U/L)**	32 (16, 84)	42 (21, 76)	0.240
**AST (U/L)**	30 (20, 54)	46 (34, 108)	<0.001
**ALP (U/L)**	101 (78, 149)	119 (87, 227)	0.049
**GGT (U/L)**	93 (38, 219)	119 (40, 212)	0.576
**LDH (U/L)**	270 (209, 386)	502 (301, 667)	<0.001
**CK (U/L)**	50 (32, 87)	137 (58, 435)	<0.001
**TBIL (μmol/L)**	16 (11, 25)	17 (11, 48)	0.129
**DBIL (μmol/L)**	6 (4, 11)	9 (5, 34)	0.006
**IBIL (μmol/L)**	9 (6, 13)	8 (5, 17)	0.852
**TC (mmol/L)**	4.25 (3.34, 5.54)	3.32 (2.34, 3.83)	<0.001
**TG (mmol/L)**	1.59 (0.99, 2.99)	1.73 (1.16, 2.83)	0.527
**TP (g/L)**	61 (57, 67)	54 (52, 61)	<0.001
**ALB (g/L)**	35.2 ± 5.0	31.5 ± 5.2	<0.001
**GLB (g/L)**	25.9 (22.6, 28.9)	22.9 (19.4, 26.9)	<0.001
**A/G**	1.35 (1.20, 1.60)	1.34 (1.10, 1.60)	0.805
**UREA (mmol/L)**	5.9 (4.3, 7.9)	11.0 (7.2, 17.1)	<0.001
**CREA (μmol/L)**	61 (50, 75)	87 (61, 163)	<0.001
**Ca (mmol/L)**	2.12 (2.00, 2.23)	2.03 (1.78, 2.13)	<0.001
**K (mmol/L)**	3.94 (3.65, 4.22)	3.86 (3.68, 4.29)	0.990
**Na (mmol/L)**	138.3 (136.0, 140.4)	140.9 (136.7, 145.1)	0.004
**Cl (mmol/L)**	103.0 (100.3, 105.6)	104.8 (101.3, 108.0)	0.026
**GLU (mmol/L)**	6.9 (5.3, 9.2)	8.0 (5.3, 9.5)	0.186
**UA (μmol/L)**	263 (189, 348)	259 (197, 329)	0.847
**AMY (U/L)**	309 (97, 866)	679 (122, 1,475)	0.053
**uAMY (U/L)**	2,299 (658, 5,431)	4,459 (1,425, 6,323)	0.042
**PTAR**	0.033 (0.029, 0.038)	0.042 (0.035, 0.050)	<0.001

**Note:**

Data are expressed as number, mean ± standard deviation, or median (interquartile range). Abbreviations: CRP, C-reactive protein; WBC, leukocyte; LY, lymphocytes; MO, monocytes; NE, neutrophils; RBC, red blood cell; HGB, hemoglobin; HCT, hematocrit; MCV, mean corpuscular volume; MCH, mean corpuscular hemoglobin; MCHC, mean corpuscular hemoglobin concentration; RDW, red cell distribution width; PLT, platelets; PCT, thrombocytocrit; MPV, mean platelet volume; PDW, platelet distribution width; PT, Prothrombin time; PT-INR, prothrombin time-international normalized ratio; APTT, activated partial thromboplastin time; FIB, fibrinogen; TT, thrombin time; AFP, alpha fetoprotein; CA199, carbohydrate antigen 199; ALT, alanine aminotransferase; AST, aspartate aminotransferase; ALP, alkaline phosphatase; GGT, γ-glutamyl transpeptidase; LDH, lactate dehydrogenase; CK, creatine kinase; TB, total bilirubin; DBIL, direct bilirubin; IBIL, indirect bilirubin; TC, total cholesterol; TG, triglycerides; TP, total protein; ALB, albumin; GLB, globular proteins; UREA, urea nitrogen; CREA, creatinine; Ca, calcium; K, potassium; Na, sodium; Cl, chlorine; GLU, glucose; UA, uric acid; AMY, serum amylase; uAMY, urinary amylase; PTAR, prothrombin time-international normalized ratio to albumin ratio.

### Independent predictors of 30-day mortality in patients with AP

**Figure 1:**
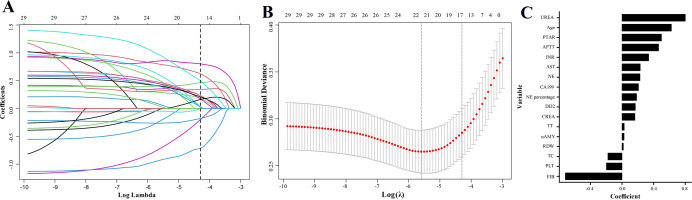
Feature selection using LASSO regression. (A) Coefficient profile diagram showing the trajectories of coefficients for each candidate variable as the tuning parameter λ varies. (B) Cross-validation curve for LASSO regression. The optimal λ was determined using the one-standard-error rule (lambda.1se). (C) Final set of potential predictors selected by LASSO regression using lambda.1se.

We first transformed these continuous factors in Table 2 with *P* < 0.1 into binary variables, to avoid collinearity, Lasso regression analysis was applied. Finally, seventeen binary variables were selected: UREA, age, PTAR, APTT, INR, AST, NE count, CA199, NE percentage, D-dimer, CREA, TT, uAMY, RDW, TC, PLT, and FIB (Fig. 1). Then, the univariate and multivariate logistic regression analyses were used further to screen independent predictors. As shown in Table 3, seven variables were proven to be independent prognostic factors: Age: hazard ratio (HR) 2.946, 95% confidence interval (CI) [1.427–6.082], *P* = 0.003; NE percentage: HR 3.364, 95% CI [1.573–07.194], *P* = 0.002; PLT: HR 0.350, 95% CI [0.141–0.868], *P* = 0.024; FIB: HR 0.157, 95% CI [0.064–0.386], *P* < 0.001; AST: HR 2.587, 95% CI [1.229–5.447], *P* = 0.012; UREA: HR 3.595, 95% CI [1.657–7.799], *P* = 0.001; PTAR: HR 7.994, 95% CI [2.339–27.327], *P* = 0.001.

**Table 3:** Univariate and multivariate logistic regression analyses for factors associated with 30-day mortality.

Variables	Univariate analysis			Multivariate analysis			
	OR	95% CI	*P*	β	OR	95% CI	*P*
**Age (years)**	5.027	[2.791–9.713]	<0.001	1.080	2.946	[1.427–6.082]	0.003
**NE percentage (%)**	3.754	[2.013–7.000]	<0.001	1.213	3.364	[1.573–7.194]	0.002
**PLT (×10** ^**9**^ **/L)**	0.181	[0.080–0.410]	<0.001	−1.051	0.350	[0.141–0.868]	0.024
**FIB (g/L)**	0.183	[0.093–0.362]	<0.001	−1.851	0.157	[0.064–0.386]	<0.001
**AST (U/L)**	4.490	[2.309–8.730]	<0.001	0.951	2.587	[1.229–5.447]	0.012
**UREA (mmol/L)**	9.683	[4.799–19.538]	<0.001	1.280	3.595	[1.657–7.799]	0.001
**PTAR**	15.592	[2.590–50.762]	<0.001	2.079	7.994	[2.339–27.327]	0.001
**AUC**	0.914						
**Clinical model**	Logistic P = 1.080 × Age + 1.213 × NE percentage − 1.051 × PLT − 1.851 × FIB + 0.951 × AST + 1.280 × UREA + 2.079 × PTAR − 8.535						

**Note:**

Abbreviations: NE, neutrophils; PLT, platelets; FIB, fibrinogen; AST, aspartate aminotransferase; UREA, urea nitrogen; PTAR, prothrombin time-international normalized ratio to albumin ratio; AUC, area under the curve.

### Establishment and internal validation of the model

**Figure 2:**
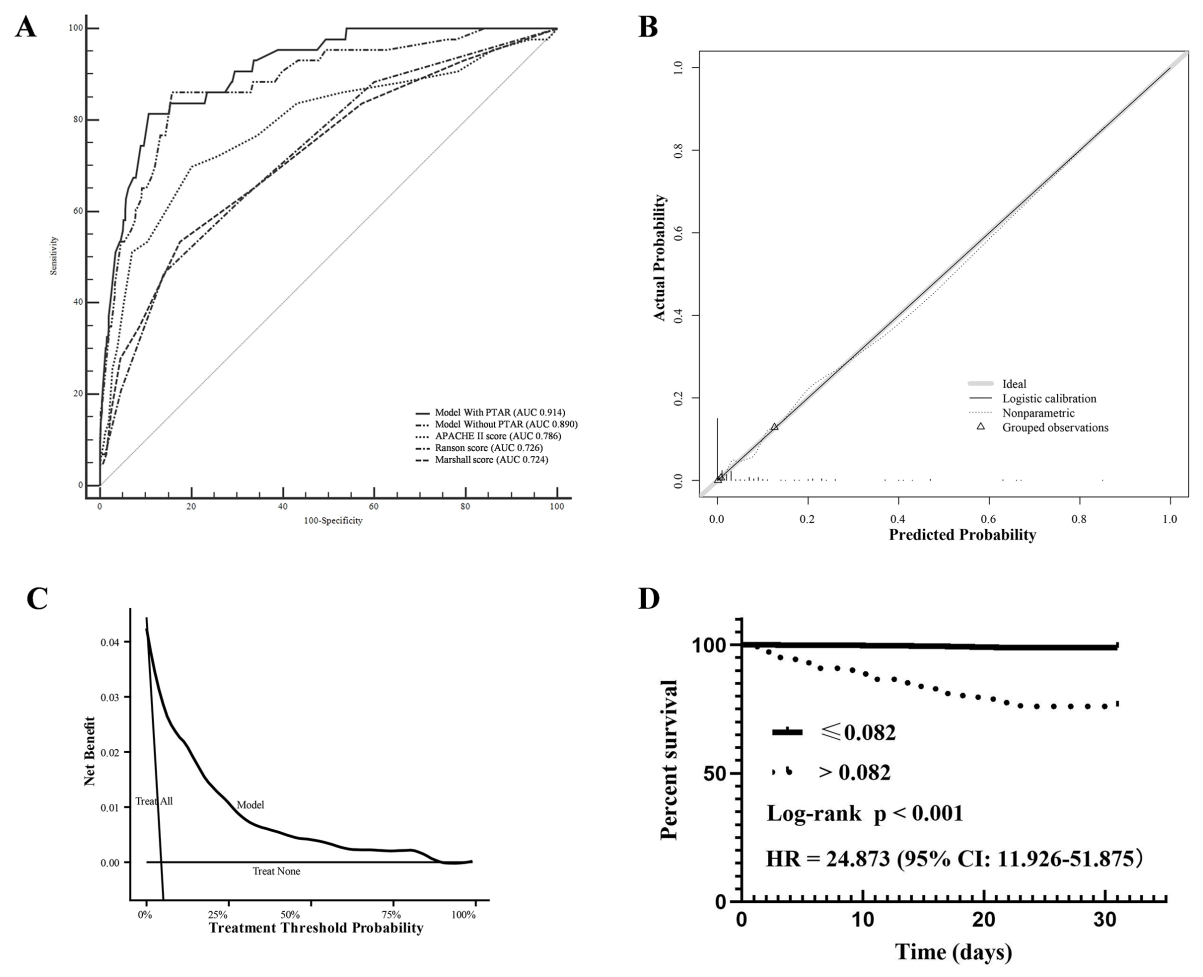
Performance evaluation of the prediction model for 30-day mortality. (A) Receiver operating characteristic (ROC) curves. The ROC curves illustrate the discriminatory ability of different models. Higher AUC indicates better discrimination. (B) Calibration curves. Calibration curves assess the agreement between predicted probabilities (x-axis) and observed outcomes (y-axis). (C) Decision curve analysis (DCA).The “treat all” and “treat none” lines serve as reference strategies. A model is considered clinically useful if it yields higher net benefit than these reference strategies across clinically relevant threshold probabilities. (D) Kaplan-Meier survival curves for 30-day mortality. Patients in the high-risk group (predicted risk > 0.082) demonstrated significantly lower survival compared to those in the low-risk group (predicted risk ≤ 0.082) (log-rank *P* < 0.001).

A prognostic model was established based on the seven independent risk factors, constructed as follows: Logit P = 1.080 × Age + 1.213 × NE percentage − 1.051 × PLT − 1.851 × FIB + 0.951 × AST + 1.280 × UREA + 2.079 × PTAR − 8.535. As shown in Fig. 2A, the model including PTAR had the highest AUC of 0.914 (95% CI [0.877–0.951]). When PTAR was excluded from the prognostic model, the AUC decreased to 0.890 (95% CI [0.869–0.909]). The AUCs for other traditional models, namely APACHE II score, Ranson score, and Marshall score, were 0.786 (95% CI [0.750–0.812]), 0.726 (95% CI [0.696–0.754]), and 0.724 (95% CI [0.694–0.752]), respectively. Furthermore, the PTAR model had the best predictive performance with sensitivity 79.1% and specificity 88.3%, and was a better prediction model (Table 4). It demonstrated a PPV of 26.3% and a NPV of 99.0%. The calibration curves revealed good predictive accuracy of the model (Fig. 2B). The calibration curves and decision curve analysis showed that this model provided a significant additional net clinical benefit to AP patients in predicting 30-day mortality, demonstrating good clinical applicability and effectiveness (Fig. 2C). The internal validation indicated good discriminatory ability of the model. In addition, K-M survival curve analysis indicated that AP patients with the value >0.082 were significantly associated with an increased risk of 30-day mortality compared to those with ≤0.082 (log-rank *P* < 0.001). Univariable Cox regression demonstrated that the value >0.082 was associated with a significantly increased risk of mortality (HR = 24.873, 95% CI [11.926–51.875], *P* < 0.001) (Fig. 2D). Therefore, clinicians can use our model to identify high-risk populations.

**Table 4:** Predictive performance comparison between the clinical prediction model with PTAR, the model without PTAR, and traditional scoring systems.

	AUC (95% CI)	Sensitivity	Specificity	PPV	NPV	*P*
**With PTAR**	0.914 [0.877–0.951]	79.1%	88.3%	26.3%	99.0%	Reference
**Without PTAR**	0.890 [0.869–0.909]	83.7%	83.5%	20.3%	99.2%	0.046
**APACHE II score**	0.786 [0.750–0.812]	69.8%	79.9%	14.0%	98.3%	0.002
**Ranson score**	0.726 [0.696–0.754]	46.5%	86.0%	13.4%	97.2%	<0.001
**Improved Marshall score**	0.724 [0.694–0.752]	53.5%	82.4%	12.4%	97.4%	<0.001

**Note:**

Abbreviations: PTAR, prothrombin time-international normalized ratio to albumin ratio; AUC, area under the receiver operator characteristic curve; CI, confidence interval; APACHE II Score, Acute Physiology and Chronic Health Evaluation II Score; PPV, positive predictive value; NPV, negative predictive value.

## Discussion

Owing to the severe complications associated with AP, the mortality rate remains high, imposing a substantial economic burden on society and family (Krishna et al., 2017). Accurately predicting the risk of mortality in AP patients is crucial for guiding clinical management and improving prognosis. Traditional scoring systems have inherent limitations, and predictive models derived from these systems and parameters often demonstrate suboptimal accuracy (Capurso et al., 2023). Recently, numerous studies have focused on creating prognostic models for AP. Gao et al. (2024) developed a web calculator using a prediction model based on six indicators to predict the risk of persistent organ failure of patients with AP. Li et al. (2023) established a nomogram incorporating seven laboratory indexes to predict the progression for severe acute pancreatitis (SAP). However, there remains a shortage of validated biomarkers to effectively assess the risk of poor prognosis in patients with AP. Therefore, this study aimed to construct a prognosis model for AP based on PTAR and other multi-source data to improve the accuracy of predicting the severity and clinical outcomes, thereby providing more robust decision support for clinicians.

We evaluated potential factors as independent prognostic predictors. To address potential multicollinearity among laboratory variables, we used LASSO analysis and multivariate logistic regression for variable selection. This resulted in a new model incorporating age, NE percentage, PLT count, FIB, AST, UREA, and PTAR levels. The model demonstrated superior performance in accurately assessing 30-day mortality in AP patients compared to traditional scoring systems, with a good AUC of 0.914.

Recent studies have found that several of these factors are related to the development of AP. Age is a well-recognized prognostic factor. Gu & Liu (2025) established an XGB model containing age to predict short- and long-term AP prognosis by collecting the dynamic variables of patients during their hospitalization. Simultaneously, the results of Ning’ study demonstrated that age ≥50 years was a key factor associated with mortality in patients with IPN (Ning et al., 2025). Our data confirmed that age was an independent risk factor for 30-day mortality, likely due to diminished physiological reserve, impaired immune function, reduced tissue regeneration capacity, and higher prevalence of malnutrition in older adults (Gardner et al., 2008).

Neutrophils and platelets are central to the inflammatory response in AP. NE percentage, a marker of systemic inflammation and physiological stress, offer a more nuanced view of the immune response than the total WBC count (Gomez et al., 2008; Gibson et al., 2007) and better reflects complications like necrosis or organ failure (Azab et al., 2011). Furthermore, platelet activation played an important role of in the pathogenesis of AP and the development of complications in S-AP (Uhlmann et al., 2008; Osada et al., 2012). Reduced platelet count indicated severe course of pancreatitis (Mariyko, Shlyakhova & Mariyko, 2022). Consistent with these findings, we observed a significant increase in neutrophil percentage and a decrease in platelet count in non-survivors, which may indicate persistent inflammation and lead to poor prognosis.

UREA reflects protein metabolism, nutritional status and renal function. The elevated level is associated with underlying catabolism in acute illnesses, including AP, acute myocardial infarction, and COVID-19 infection (Faisst et al., 2010; Cheng et al., 2020; Zhu et al., 2022). As an important component of the liver enzyme profile, AST not only reflects the degree of liver involvement in AP, but also serves as a significant biomarker for predicting the severity and prognosis of the disease. AST > 250 U/L is one of the five indicators in the Ranson score at admission. Studies have shown that a persistently elevated AST in SAP patients is positively correlated with the risk of pancreatic necrosis and organ failure (Hu et al., 2023). Yuan et al. (2022) describes a machine learning framework based on ubiquitously available clinical variables and confirmed the value of age, Urea and AST in accurately predicting the development of AP. Moreover, evidence showed that the level of AST was significantly higher in AP patients with a Ranson score ≥3 (Abaylı, Gençdal & Değirmencioğlu, 2018), and early elevation of creatinine within 24 h after admission was a good predictor of death in patients with AP (Wan et al., 2019). In accordance with the findings of prior studies, our research showed that UREA and AST levels were correlated with patients’ prognostic status.

FIB is a key component of the coagulation and fibrinolytic systems. The increase in FIB levels reflects the enhancement of fibrinolytic activity in the body and plays a fundamental role in the process of venous thrombosis, which can be significantly elevated (Pang et al., 2021). Previous studies have shown that systemic hypercoagulable state or pre-thrombotic state is closely related to the prognosis of patients with AP (Zhou et al., 2015). Zheng et al. (2023) reported that FIB was a significant independent risk factor with high predictive value for severe complications in patients with SAP. Wang et al. (2025) demonstrated that FIB may be a useful biomarker for assessing the severity of hyperlipidemic AP. Similarly, we found a higher FIB level in the nonsurvivors. Patients with AP may also have liver function disorder and reserves damage (Trikudanathan et al., 2024). Both INR and albumin are synthetic indicators of liver. PTAR, derived from INR and albumin, might more comprehensively reflect the liver function. Elevated PTAR levels are significantly correlated with poor prognosis of various diseases (Wang et al., 2021), which is also supported by our study. We determined an optimal PTAR cut-off value and found that a higher PTAR was associated with better survival, indicating its utility as a sensitive and specific indicator for short-term outcomes in AP. Combining PTAR with other indicators significantly enhanced prognostic predictive value.

The proposed model had several advantages. First, the blood markers included in the predictive model were readily tested as part of the admission examinations, with no additional costs. Compared with the currently recommended scoring systems including Ranson criteria, APACHE II, and Marshall score, the PTAR-based model is simpler and effective for prognosis prediction. In addition, since our model only includes objective indicators of disease deterioration, its reproducible prediction of short-term mortality in patients with AP is reliable. We reweighted the predictive impact of each indicator to improve the predictive performance of the model. For clinical application, we suggest a stepwise approach. Clinicians can calculate an individual patient’s predicted probability using the model, stratify patients into risk categories, and consider those with a probability >0.082 as high risk for 30-day mortality. High-risk patients should receive early intensive monitoring, including frequent vital sign assessments, more frequent cross-sectional imaging with either computed tomography (CT) or magnetic resonance imaging (MRI), consideration for higher-acuity care, prompt nutritional support, and aggressive fluid resuscitation as indicated.

This study has several limitations. First, its retrospective design introduces potential selection and information bias. Although strict inclusion criteria and interpolation methods for missing data were applied, data quality issues may still affect the model’s accuracy and reliability. The results may not be generalizable to other centers or populations. Second, although the overall sample size was substantial, the number of patients with poor outcomes was limited, which may affect the generalizability of the findings. Third, while the model performed well in internal validation, it lacks external validation, limiting its applicability to other settings. To address this, we plan a larger multicenter prospective study for external validation and potential refinement. Finally, due to data limitations, we could not perform subgroup analyses based on AP etiology (*e.g*., gallstones, hypertriglyceridemia, alcohol). As etiology may influence disease trajectory, future studies should evaluate the model’s performance across different etiological subgroups.

## Conclusion

We developed an interpretable and widely applicable model based on age, NE percentage, PLT count, FIB, AST, UREA, and PTAR. This model demonstrated superior accuracy compared to traditional scoring systems in predicting 30-day mortality in AP patients. It may serve as a useful supplement to standard approaches, improving outcome predictions and supporting effective management.

## Supplemental Information

10.7717/peerj.21415/supp-1 Supplemental Information 1 Raw Data. Clinical parameters and laboratory data of all participating patients with acute pancreatitis.

10.7717/peerj.21415/supp-2 Supplemental Information 2 STROBE Documentation.

## Additional Information and Declarations

### Data Availability

The following information was supplied regarding data availability:

The data is available in the Supplemental File.
